# Risk Denial and Socio-Economic Factors Related to High HIV Transmission in a Fishing Community in Rakai, Uganda: A Qualitative Study

**DOI:** 10.1371/journal.pone.0132740

**Published:** 2015-08-26

**Authors:** Muhamadi Lubega, Neema Nakyaanjo, Sumaya Nansubuga, Edgar Hiire, Godfrey Kigozi, Gertrude Nakigozi, Tom Lutalo, Fred Nalugoda, David Serwadda, Ronald Gray, Maria Wawer, Caitylin Kennedy, Steven James Reynolds

**Affiliations:** 1 Rakai Health Sciences Program, Old Bukoba road, P.O Box 279, Kalisizo, Uganda; 2 Department of Public Health Sciences/Global Health (IHCAR), Karolinska Institutet, 171 77, Stockholm, Sweden; 3 School of Graduate Studies and Research Busoga University, PO BOX 154, Iganga, Uganda; 4 Division of Intramural Research, National Institute of Allergy and Infectious Diseases, National Institutes of Health, Bethesda, Maryland, and Johns Hopkins University School of Medicine, Baltimore, Maryland, United States of America; 5 Johns Hopkins University, Bloomberg School of Public Health, Baltimore, Maryland, United States of America; International AIDS Vaccine Initiative, UNITED STATES

## Abstract

**Background:**

In Kasensero fishing community, home of the first recorded case of HIV in Uganda, HIV transmission is still very high with an incidence of 4.3 and 3.1 per 100 person-years in women and men, respectively, and an HIV prevalence of 44%, reaching up to 74% among female sex workers. We explored drivers for the high HIV transmission at Kasensero from the perspective of fishermen and other community members to inform future policy and preventive interventions.

**Methods:**

20 in-depth interviews including both HIV positive and HIV negative respondents, and 12 focus-group discussions involving a total of 92 respondents from the Kasensero fishing community were conducted during April-September 2014. Content analysis was performed to identify recurrent themes.

**Results:**

The socio-economic risk factors for high HIV transmission in Kasensero fishing community cited were multiple and cross-cutting and categorized into the following themes: power of money, risk denial, environmental triggers and a predisposing lifestyle and alcoholism and drug abuse. Others were: peer pressure, poor housing and the search for financial support for both the men and women which made them vulnerable to HIV exposure and or risk behavior.

**Conclusions:**

There is a need for context specific combination prevention interventions in Kasensero that includes the fisher folk and other influential community leaders. Such groups could be empowered with the knowledge and social mobilization skills to fight the negative and risky behaviors, perceptions, beliefs, misconceptions and submission attitudes to fate that exposes the community to high HIV transmission. There is also need for government/partners to ensure effective policy implementation, life jackets for all fishermen, improve the poor housing at the community so as to reduce overcrowding and other housing related predispositions to high HIV rates at the community. Work place AIDS-competence teams have been successfully used to address high HIV transmission in similar settings.

## Introduction

Uganda has significantly reduced the adult HIV prevalence from a peak 18% in 1992 to 7.3% in 2011. [1,2] In Rakai district, South-Western Uganda, however, HIV prevalence and incidence remain significantly higher than in the other parts of the country. In Kasensero fishing community in Uganda, home of the first recorded case of HIV in Uganda, HIV transmission is still very high with an incidence of 4.3 vs 3.1 per 100 person-years in women and men, respectively, and an HIV prevalence of 44%, reaching up to 74% among female sex workers.[3] Studies in nine other sub-Saharan African countries have shown that fishing communities have higher incidence rates and also HIV prevalence rates that are 4 to 14 times greater than in the general population.[4,5,6]. The high HIV transmission in fishing communities has been attributed to the contextual socio-economic vulnerabilities and the lack of prevention and care services in these settings[6]. UNAIDS and the WHO emphasized the need to understand the contextual dynamics of local HIV transmission modes as a foundation for developing effective locally targeted combination HIV prevention strategies. [7,8,9] This study was therefore aimed at obtaining an in-depth understanding of the underlying contextual drivers of the high HIV rate in Kasensero from the perspective of the community itself for effective policy and preventive intervention formulation. [10] [11]

## Materials and Methods

### Study Setting and population

Between April and September 2014, we conducted this study at the Kasensero fishing community, Rakai district, south western Uganda approximately 200 km from the capital city Kampala. The community constitutes a population of approximately 15,000 people. The site is also connected to five other smaller fishing communities many of which attract both Ugandan and Tanzanian fisher folk. The majority of the population is mobile. Kasensero directly or indirectly depends on fishing. The community consists of fisher folk, fish processors, boat builders, fishing gear makers/repairers, retail traders and other casual income earners. The fisher folk consist of boat owners, boat crew members and the fish traders who buy fish for either wholesale or retail markets. Most people reside in temporary houses, some occupied by more than one family. There are over 40 lodges (hotels) and 80 bars open 24 hours a day though out the week with young women whose main source of income is commercial sex work. There is also a large fish factory which processes and freezes fish, and there is heavy traffic of freezer trucks which convey the frozen catch for export to Europe. All these groups have different contextual patterns of interactions, access to money and patterns of seasonal migration and engagement in sexual relationships that may affect their vulnerability to HIV acquisition.

The community is served by two outpatient health facilities offering HIV testing and counseling (HTC), pre-antiretroviral (ARV) care, CD4 monitoring as well as antiretroviral therapy (ART) services. There are ten drug shops/private clinics which are not officially accredited to offer ART but often sell cotrimoxazole, condoms and contraceptives all of which are important components of combination HIV prevention. The prevalence of HIV is 44.3% (74.5% among female bar workers), and HIV incidence is 4.3/100 person-years in women and 3.1/100 person-years in men [12].

This qualitative study employed 12 focus group discussions (FGDs) and 20 in depth interviews (IDIs) with HIV infected and uninfected persons aged 15 years and older from the Kasensero community to explore possible drivers of the high HIV transmission in this population (Tables 1 and 2).[13] The FGD and IDI respondents were purposively selected from a sampling frame designed to elicit different viewpoints [13].The respondents were selected because they were considered to be more “knowledge rich” on the study subject in their own situations than anybody else [13]. The participants were chosen from consenting members of the Rakai Community Cohort Study (RCCS) all of whom knew their HIV status from previous surveys by the RHSP. The FGDs were stratified by gender and included HIV infected and uninfected respondents to avoid potential stigmatization. Participants were invited to take part in only one FGD or IDI. Each FGD consisted of a maximum of 12 participants. While each of the participants knew their HIV status, the participants were not told how their groups were selected or their sero-status to avoid “playing to type”

**Table 1:** General characteristics of in depth interviewees (IDIs) (n = 20). F Female. M Male

No	Age	Sex	Education	Religion	M/ status	Occupation	(RCCS) Category
1	27	F	Primary	Catholic	Single	Bar owner	Sex worker HIV negative
2	35	F	None	Moslem	Single	Bar owner	Sex worker HIV positive
3	17	F	Primary	Born again	Single	Fish trader	HIV negative female 15–19
4	37	F	Primary	Catholic	Single	Fish trader	HIV positive female 35yrs plus
5	39	M	Primary	Adventist	Single	Fishing	HIV positive fishermen
6	41	M	Primary	Catholic	Single	Fishing	HIV positive fishermen
7	49	M	None	Catholic	Single	Fishing	HIV negative fishermen
8	41	M	None	Moslem	Single	Fishing	HIV positive men 35yrs plus
9	16	M	Secondary	Moslem	Single	Student	HIV positive men 15–19
10	21	F	Primary	Born again	Married	Housewife	HIV positive female 20–34yrs
11	23	M	Primary	Catholic	Single	Fishing	HIV positive men 20–34yrs
12	20	F	Primary	Protestant	Married	House wife	HIV negative female 20–34yrs
13	19	M	Secondary	Catholic	Single	Boda-boda rider	HIV positive men 15–19 yrs
14	25	F	Primary	Protestant	Single	Sex worker	Sex worker HIV negative
15	39	M	None	Moslem	Single	Fishing	HIV negative men 35yrs plus
16	22	M	Primary	Catholic	Married	Mechanic	HIV negative men 20–34yrs
17	38	M	Secondary	Moslem	Married	Fishing	HIV negative fishermen
18	28	F	Secondary	Protestant	Married	Tailor	Sex worker HIV positive
19	38	F	Secondary	Born again	Married	Peasant	HIV negative female 35yrs plus
20	19	F	Primary	Catholic	Married	House wife	HIV positive female 15–19 yrs

**Table 2:** General characteristics of FGD participants (n = 92). F Female. M Male

FGD No	Respondents	Sex	Age range	Participants’ category	Duration
1	06	F	15–19	HIV positive females 15–19 years	74 min
2	12	M	38–49	HIV positive fishermen	75 min
3	09	M	38–49	HIV negative fishermen	75 min
4	07	M	15–19	HIV negative men 15–19 years	80 min
5	08	F	20–38	HIV positive bar maids/sex workers	67 min
6	06	M	35–39	HIV positive men 35 years and above	69 min
7	07	M	35–39	HIV positive females 35 yrs and above	73 min
8	07	F	25–45	HIV positive bar maids/owners (sex workers)	75 min
9	09	M	20–34	HIV positive men 20–34 years	75 min
10	08	F	20–34	HIV positive females 20–34 yrs	49 min
11	07	F	19–45	HIV negative female fish traders	64 min
12	06	F	25–65	HIV positive female fish traders	42 min

### Data collection tools and methods

Using a topic guide, the selected participants were probed on their views about social and economic drivers of high HIV transmission, socioeconomic expectations in sexual relationships by both men and women and how the socio-economic status of men, and women in this fishing industry predisposed them to HIV risk. The guide also captured the respondent’s individual and family background prior to the interview, their social networks and how they affected individual and group behavior towards CHP prevention and HIV risky sexual behaviors. Interviews stopped when it was judged that the point of saturation had been reached and no more new information could be retrieved from the respondents.

All data collection was supervised and assessed by the first author (ML) who is a male, indigenous public health physician and HIV scientist and the second author (NN) who is a social scientist with experience in qualitative research in the study setting for over five years. Five research assistants moderated and took notes for the study. The research assistants were conversant with qualitative data collection methods, had been working with RHSP for over five years, had conducted similar research in the study setting and were fluent in Luganda, (the local language). They were trained for two days on the study aim, design and tools. Role-plays were used to prepare the research assistants for their interaction with the informants [14,15]. The experiences from the role-plays were discussed and further methodological guidance was given. All the FGDs and the IDIs were conducted in Luganda, transcribed and later translated into English by the interviewers. The authors listened to the tapes to confirm the validity of the information. Data collection stopped when information relating to the topic guides revealed no new information.

### Data analysis

Data analysis was iterative including reviews and discussions at different stages of data collection and appropriate modifications were made in the tools to address emerging issues [16,17]. The units of analysis were the transcripts from the IDIs and the FGDs. Content analysis was used to analyze the transcripts. This entailed reading and reviewing texts of the entire interview back and forth to identify meaningful units in relation to the study subject[16,17,18,19]. The meaningful units were condensed and coded by categories and themes, and discussed until consensus was reached on the appropriate codes and the themes[17].

### Ethical clearance

The study was approved by the Uganda Virus Research Institute (UVRI) Science and Ethics Committee (SEC), and the Uganda National Council for Science and Technology (UNCST). We also sought the approval of the district authorities in Rakai district. As part of the informed consent, the participants were told about the aims of the study, the anticipated benefits and risks, their ability to participate or withdraw at any time, and assured that all information obtained would be kept confidential. For the minors, written informed consent for participation was sought from their guardians or next of kin who signed on their behalf. The participants signed two copies of the consent form before the interview commenced, and one copy was given to the participant.

## Results

Our analysis of textual data generated six themes including: (1) the power of money, (2) risk denial, (3) predisposing environment and lifestyle, (4) peer pressure, (5) poor housing and (6) the search for social/material support. We describe each of these themes in detail below.

### The power of money

From the accounts of the respondents, money was often reported as the driver for the high HIV transmission in Kasensero influencing or influenced by migration patterns, sex starvation and what they termed easily disposable income

#### Migration patterns

Because the fish migrated from one site to another, A few fisher men also reportedly moved to these sites in search for the fish which predisposed them to multiple sex partners whose sero-status was unknown.

“You know, we migrate with the fish from one small site to another depending on where the big catch is …… In so doing, we get exposed to many women some of who may be HIV positive…” (Male informant, 41 years, HIV positive fisherman)

#### Sex starvation

As well, most of the young fishermen reported spending 4–7 days on the lake catching fish and were by their own definition “sexually starved.” when they came back to the main land, they reportedly had as much sex as possible which, in their view, predisposed them to HIV acquisition.

“Some of us spend days or even a week there (on the lake) without any sex at all yet this is a basic natural thing. So when we come back, we have to compensate, so I try to have as much sex as possible. Even three to four women in a day…so this also predisposes us to a high risk of acquiring HIV." (Male informant, 41 years HIV positive fisherman)

#### Easily disposable income

A few fishermen reported that they caught much fish throughout all seasons, required very little material input to do their job, and found little incentive to save money. They earned large sums of money and were willing to spend it immediately and lavishly on sex. This was cited as increasing their vulnerability for HIV acquisition.

“I can’t deny that I don’t love sex, and remember here once you have money you will have sex with any woman you want. I sell my fish and buy a woman of my choice or even two…… then I go back in the evening because the lake is not drying, the fish is always there so I have a permanent daily income forever, so why not make myself happy.” (Male informant, 39 years HIV positive fisherman)

“If he wants a woman, he can buy her for sex with any amount of money because he has the money and earns that money every day. So he does not feel like he is wasting that money because he will earn the same amount of money the next day. He just has to sit in the boat and he earns the money.” (Female HIV positive, 15–19 years FGD)

### Risk denial

Lack of HIV related fear was frequently reported by many respondents as a determinant for the high HIV transmission in Kasensero and this was due to the much higher and outcompeting risk to die from drowning. Most respondents reported seeing their colleagues die during lake storms more often than HIV related deaths and therefore perceived the risk of dying on the water as high, not knowing whether they would survive the next day. They therefore perceived work related mortality as a much greater than their risk of dying from HIV.

“Here almost every day people die in the lake either because of storms or boats sinking. We lose like two to three of our friends every week, so the main question we ask ourselves is why waste time investing in life, instead of having good sweet unprotected sex, that you are fearing HIV…… after all you may fear HIV but still the lake will swallow (sink to death)you. So for us we just enjoy life man.” (Male informant, 41 years fishermen HIV positive)

### Predisposing environmental and lifestyle factors

This theme included many bars/ lodges, daily social events/pornography, alcohol and drug abuse, redundancy, and ineffective policies

### Many bars and lodges

The area of study has an abundance of alcohol serving venues open 24/7 and accompanying lodges that offer cheap over-night accommodation. The majority of the men reported being drunk almost constantly during their free time. The drunkenness reportedly increased the fishermen’s sexual desires, and impaired judgment on when to have unprotected sex while on the shores. This in their view was complimented by the many cheap lodges available which provided venues for sex encounters at any time of the day with the readily available sex workers

“There are so many bars and lodges, over 80 in this place and they work all the time. Many bars here actually also act as lodges in the behind quarter with many beautiful brown girls, so you drink, and then feel like having sex, so you get one, buy her some beer and pay her for sex, protected or unprotected depending on what you want and how much you can offer.” (HIV positive men, FGD)

#### Daily social events and pornography

A few respondents reported that there were also many daily social events in Kasensero such as music festivals and film shows almost every day. These events were reported to be hubs for sexual exposure because they brought people of all classes together, to watch sex films, drink alcohol and provide access to commercial sex.

“Kasensero has many places for fun; many bars, discos and clubs. There is limited space for one to avoid HIV....Okay when people go to these events, remember there are so many beautiful women, almost half naked, so they buy them alcohol etc, they all become drunk and excited, so even if one is to protect yourself they end up forgetting or getting overpowered by the desire and hence have un protected sex. I think it is one reason HIV will always be here in Kasensero.” (HIV negative, 35years and above female FGD)

“You know doctor, (referring to the moderator), there are so many areas here with cinema halls showing sex films. So many of us when we come back from the lake, remember we are loaded with our money, we spend time here watching and learning several styles of sex as an appetizer……and even the sex workers know, they wait for us here in these halls, so after watching the movie together, what next obviously we go for sex and you want to enjoy it the way you saw it in the film. It’s sweet life you know…” (Male informant, 39 years HIV positive fisher man)

#### Alcoholism and drug abuse

Some respondents also attributed the high HIV transmission in Kasensero to excessive alcohol and drug abuse that were rampant, especially among the fishermen and sex workers. In their views intoxication with alcohol or drugs such as Marijuana and kuba either increased the people’s libido, or made them lose judgment on when or even whether to have protected sex.

“You know the drugs or alcohol changes their brains and behaviors, they become fearless, even the women, the moment they are drunk or after drugs, they feel they want sex. So it is cheaper for us then because they will accept any amount when they are charged. “HIIV positive fishermen FGD)

“Sometimes when there is less fish at the site, the fishermen have little money and they don’t buy us expensively. You have to sleep with many men (5–10) in a day to earn a living so sometimes we have to take alcohol or drugs (marijuana) so we have the courage to do this or even not to feel the psychological pain. This also spreads HIV.” (Female informant, 28 years, barmaid/sex worker)

#### Redundancy

A few respondents attributed the high HIV transmission to redundancy especially among the fishermen during day-time when they come back from the lake or at night when they do not have anything to do but socialize. In their perception, the only activity was getting a sex partner to spend time with.

“Most fishermen here work at night so during day they are redundant so they choose to have fun. All women here know it very well those fishermen always have their money at hand. Attractive girls walk to these men and fishermen cannot hesitate to ask for sex………………they have a lot of time to hangout”. (HIV negative fishermen FGD)

#### Ineffective local policy implementation

The community also reportedly had weak or ineffective local policy implementation to prevent HIV transmission either because of a permissive or corrupt police in addition to weak community leadership against a community that lacked any norms of social control for CHP.

The policemen are our friends, by the way even the local leaders, you look at me, who of those people would not like to have sex with a beauty like me, so instead of arresting us and harassing us we have just become sex pals (sex friends). Often they come and we offer them free sex as long as we don’t publicize it.”(Female informant, 27 years bar owner/sex worker)

### Peer pressure

From the accounts of a few respondents, peer pressure ranged from age mates especially 15–19 years or friends enticing others into having early sex, having sex for money or having unprotected sex for more money as a better way to live and make quick money without suffering.

“I used to insist on abstaining from pre-marital sex but life was very hard, so my friends told me it was useless living a hard life when God gave us a gift of being a woman, so they started taking me to bars, discos etc, eventually I decided to join them in having sex for money. …….and for the two years I have been here my life has changed positively. I earn a lot of money from selling my body (sex trade) l”. (HIV positive, 15–19 years female FGD)

“Sometimes a friend will tell you how good or sweet a given woman is especially if you have natural (unprotected) sex, so when more than one person praises a given woman, you also get the drive to have unprotected sex with her and feel the same. So many of us in the same network end up having unprotected sex with the same woman and I think this also predisposes us to HIV”. (HIV negative fishermen FGD)

### Poor Housing

Most respondents intimated that housing comprised of make shift shelters made of timber and occupied by two or more families of different backgrounds and their children. Sex partners under these circumstances lacked privacy and had sexual encounters while others were listening. In some instances, fishermen reportedly left their sex partners in these public houses with other single male occupants as they went out at night for fishing which in their view could easily tempt the remaining adults to have sex without using a condom.

“Like us the fishermen, you go out at night, leave your partner in the house with another unmarried man and both of them know you are not coming back, so automatically they will have sex. Unfortunately, some even do it when the children are still awake, so you come back and the child tells you the man was sleeping on mummy and mummy was crying (having sex), the question is, where will they have gotten a condom to use at that time in the night. In revenge if you know any woman this man loves, you also look for her and pay her any money to have sex with her. I think this is one of the ways HIV is spreading among us”. (Male informant, 49 years HIV negative fisherman)

“For us we stay about six in the same room. Your room mate may bring their men and they have sex when you are listening, so your desire grows and eventually you either ring someone to come and you have sex or you go out around a club, lodge or bar and you also get someone to satisfy you. Surely this is how we end up sleeping with many men all the time because this happens almost every night”. (HIV positive bar maids/sex workers FGD)

### Social/material support

According to a few respondents, the dire need for social/material support especially among the women and some men reportedly made the recipients (men and women) vulnerable to HIV acquisition. In search for shelter, security, housing or a sense of belonging, young women and men reportedly had to find themselves in relationships, under which they had little power to bargain for safer sexual behavior,

“Also the other risk is that in need of the support, many young men and women here (have) to take on risky behavior such as multiple sex partners because they are needy for items like money, food, security, housing etc. This also predisposes them to HIV.” (HIV positive men 20–30yrears FGD)

## Discussion

Our findings indicate that the high HIV transmission in Kasensero is not a function of just the individual behavior of the people living at the site but a complexity of multiple ecological/cross cutting themes including; power of money, risk denial, environmental triggers, and a predisposing lifestyle. The others are peer pressure, poor housing and the search for financial support for both the men and women which deprives them of the social capital and foundation to bargain for safer sex.

The fishermen whose livelihood depends on fishing spend long periods away from home in search of fish. The other service providers including sex workers, transporters and processors also moved between landing sites, national and regional markets and fish processing factories. The social structures that mitigate HIV risk behaviors such as family pressure in stable couples were thus hard to sustain in the mobile fishing community of Kasensero. Such communities are prone to lack of social cohesion, personal and family responsibility/safety networks and disease prevention lifestyles for HIV acquisition. Instead they have a lifestyle that increases the likelihood of promiscuity and infidelity. Migratory lifestyles as a plat form for high HIV transmission in fishing communities has has also been identified in other fishing sites in Kenya and Bennin [20,21]. Indeed the Uganda HIV/AIDS action plan highlights managing migration at fishing sites as central to managing the high HIV rates in fishing populations[22].

The boat crew spend days away from home in search for fish. On shore, the fishermen do not carry along sex partners and yet depending on the type of fish they catch, some spend many days away in the lake. This could explain their “sex starvation” compensatory behaviors such as having as much sex as possible with many partners whenever they land with the fish. This situation is exacerbated by the readily available commercial sex workers and the disposable income of fishermen. The combination of easily disposable income, sex starvation and cheap transactional sex in fishing communities as a driver for high HIV transmission has been established by similar studies in Haipong, Uganda and other fishing sites of the Lake Victoria.[23,24,25,26,27]

As reported, the fishermen saw the risk of dying on the lake as much greater than the risk of dying from HIV which prompted them to take risks such as having unprotected sex with multiple sexual partners. Fishing is a hazardous occupation leading to a culture of risk denial and fatalism. The risk denial is an elaboration of how socio-cultural attitudes and beliefs or community values affect the individual or community responses to danger. The denial of the danger associated with HIV or HIV related death and emphasis on independence and fatalism has been established in other studies in Africa and Asia where fishermen were found to be reluctant in seeking or adhering to HIV combination prevention services because they perceived death at the lake as nearer and obvious compared to HIV related death [10,28,29,30,31,32,33]

Environmental triggers such as the many bars/lodges, free leisure time, alcohol/drug abuse, availability of commercial sex, daily social events and pornography are likely to give the community a leisefaire attitude towards life. Such contextual leisure time is bound to act as a hub or augment networks for treating redundancy and networks bound to end up into risky sexual encounters. This is fermented by the lack of law enforcement for deterrent risky sexual behavior and hence all the fabrics for high HIV transmission in such communities seem acceptable as was in cited in Kasensero. Environmental and behavioral factors as a predisposition to high HIV acquisition among the fisher folk and the surrounding dependant community has also been established in other studies of fishing communities in Uganda and other resource poor settings.[34,35,36,37,38]

Peer pressure and/or sexual-socio-economic related networks were a vulnerability to high HIV transmission for the Kasensero fishing community. The peer influence was likely to predispose many young men and women into indulging in HIV predisposing activities including but not limited to pre-marital sex, cross generational sex, sex with multiple partners specifically to earn a living and having un protected sex to earn more money. As well the networks could have acted as foci for identifying individuals and sites of the community that were much paying to the beneficiaries such as identifying the “rich” men and women in the community who were willing to pay money or in kind for sex and for prestige as well. The effect of peer pressure and social net working as a motivator for HIV predisposing sexual- socio-economic activities has been equally reported in similar in Africa and Asia settings. [39,40,41].

The need for financial support among women and young homeless men contributed to high HIV transmission in Kasensero. Such support includes money, shelter, transportation, food, and security. In such relationships, sexual encounters are typically hurried and often without protection. The subordinate position of women makes them vulnerable to abuse and sex exploitation by multiple sexual partners whose sero-status is unknown. The search for social and other support has been equally identified as a predisposition to high HIV vulnerability in similar resource constrained settings in Kenya, Malawi, Zambia, South Africa [38,42,43,44].[31]

We triangulated our data collection methods (FGDs, IDIs). This helped us to check for consistency and contradictions inside and across the groups and interviewees. The multidisciplinary and native research team was useful in understanding the contextual aspects relating to socio-economic factors for the high HIV transmission in Kasensero from the perspective of the community itself. We feel that the content analysis employed for this study has achieved appropriate in-depth analysis for the purpose of the study.

### Study limitations

We did not observe the socio-economic behaviors of the community members at the focal points such as the cinema halls, bars and lodges especially at night. This could have helped us assess the contextual perspective of risky sexual behavior that exposes this community to high HIV rates. The fact that the interviewees themselves lived this life and volunteered the information, however, strengthens the concept that the data derived from the interviews was objective to the topic of the study.

## Conclusions

There is a need for context specific combination prevention interventions in Kasensero that includes the fisher folk and other influential community leaders. Such groups could be empowered with the knowledge and social mobilization skills to fight the negative and risky behaviors, perceptions, beliefs, misconceptions and submission attitudes to fate that exposes the community to high HIV transmission. There is also need for government and other partners to ensure free and mandatory life jackets for all fish boat crew to reduce deaths on the lake as boat jackets have been found to save life in such contexts [45]. Authorities’ should also improve the poor housing at the community so as to reduce overcrowding and other housing related predispositions to high HIV rates at the community. Work place AIDS-competence teams, sustainable livelihood programmes, context specific government and nongovernmental originations and fishing community based organizations have been successfully used to address high HIV transmission in similar settings around the Great Lakes and other comparable communities.
